# A-π-D-π-A-Based Small Molecules for OTFTs Containing Diketopyrrolopyrrole as Acceptor Units

**DOI:** 10.3390/mi12070817

**Published:** 2021-07-13

**Authors:** Baji Shaik, Mujeeb Khan, Mohammed Rafi Shaik, Mohammed A.F. Sharaf, Doumbia Sekou, Sang-Gyeong Lee

**Affiliations:** 1 Department of Chemistry, Research Institute of Natural Science (RINS), Graduate School for Molecular Materials and Nanochemistry, Gyeongsang National University, Jinju 660-701, South Korea; shaikbaji2@gmail.com; 2 Department of Chemistry, College of Science, King Saud University, P.O. Box 2455, Riyadh 11451, Saudi Arabia; kmujeeb@ksu.edu.sa; 3 Department of Industrial Engineering, College of Engineering, King Saud University, P.O. Box 800, Riyadh 11421, Saudi Arabia; mfsharaf@ksu.edu.sa; 4 Department of Agricultural Extension and Rural Society, College of Food and Agriculture Sciences, King Saud University, P.O. Box 2460, Riyadh 11451, Saudi Arabia; 442106474@student.ksu.edu.sa

**Keywords:** organic thin film transistors, hole mobility, small molecules, donor-acceptor, diketopyrrolopyrrole

## Abstract

A-π-D-π-A-based small molecules 6,6′-((thiophene-2,5-diylbis(ethyne-2,1-diyl))bis(thiophene-5,2-diyl))bis(2,5-bis(2-ethylhexyl)-3-(thiophen-2-yl)-2,5-dihydropyrrolo[3,4-c]pyrrole-1,4-dione) **(TDPP-T)** and 6,6′-(((2,3-dihydrothieno[3,4-b][1,4]dioxine-5,7-diyl)bis(ethyne-2,1-diyl))bis(thiophene-5,2-diyl))bis(2,5-bis(2-ethylhexyl)-3-(thiophen-2-yl)-2,5-dihydropyrrolo[3,4-c]pyrrole-1,4-dione) **(TDPP-EDOT)** have been designed and synthesized. The diketopyrrolopyrrole acts as an electron acceptor, while the thiophene or 3,4-ethylenedioxythiophene acts as an electron donor. The donor–acceptor groups are connected by an ethynyl bridge to further enhance the conjugation. The optoelectronics, electrochemical, and thermal properties have been investigated. Organic thin film transistor (OTFT) devices prepared from **TDPP-T** and **TDPP-EDOT** have shown p-type mobility. In as cast films, **TDPP-T** and **TDPP-EDOT** have shown a hole mobility of 5.44 × 10^−6^ cm^2^ V^−1^ s^−1^ and 4.13 × 10^−6^ cm^2^ V^−1^ s^−1^, respectively. The increase in the mobility of **TDPP-T** and **TDPP-EDOT** OTFT devices was observed after annealing at 150 °C, after which the mobilities were 3.11 × 10^−4^ cm^2^ V^−1^ s^−1^ and 2.63 × 10^−4^ cm^2^ V^−1^ s^−1^, respectively.

## 1. Introduction

During the past decades, organic semiconductors have emerged as an alternative to inorganic semiconductors because of their superior advantages compared with their inorganic counterparts [1]. Among organic semiconductors, organic thin film transistors (OTFTs) are significant because of their many applications in fields like flexible displays [2], inexpensive radio frequency identification (RFID) tags [3], and sensors [4]. Exploiting the solution processable properties of OTFTs devices allows for them to be prepared easily at a low cost [5]. Tremendous work has been done by researchers on the synthesis of several small molecules and polymers constituting benzothiadiazole (BTD) [6,7,8], diketopyrrolopyrrole (DPP) [9,10,11,12], isoindigo (iI) [13,14,15], benzobisthiadiazole (BBT) [16], and arylene diimides [17,18,19], which have shown good mobility.

So far, OTFTs have been prepared from both polymers and oligomers. In general, polymers tend to achieve a high mobility for OTFTs. Nevertheless, the simple preparation and easy purification of small molecules makes them a better choice over polymers. So far, significant results have been reported on small molecule-based OTFTs, including anthracene [20,21,22], naphthalene [23,24], thienothiophene [25,26], and fluorene [27], which have demonstrated good mobility.

OTFT performances can be improved by tuning the molecular orbitals of the organic molecules. The most useful and effective way of modifying the energy levels of molecular orbitals of organic molecules is achieved using a donor–acceptor (DA) strategy. The donor–acceptor molecules have a low energy gap between the HOMO and LUMO levels. The common electron acceptor molecules are benzothiadiazole [28], benzoselenadiazole [29], and diketopyrrolopyrrole [30], while the common electron donor molecules are thiophene [31], 3,4-ethylenedioxythiophene [32], and thienothiophene [33]. Although many acceptor molecules have been explored for OFETs, diketopyrrolopyrrole remains one of the most versatile and widely used molecules. Diketopyrrolopyrrole (DPP) has a planar conjugated bicyclic structure, which enables efficient intermolecular π–π interactions, whereas the lactam part makes the DPP unit exhibit a strong electron-withdrawing effect. As for the donors in small molecule-based OTFTs, ethylene-based conjugated systems have gained decent attention from scientists and technologists [34]. The ethynylene bridge provides the diketopyrrolopyrroles coplanar to the central donor group, thus promoting extensive π-conjugation [35]. It also enhances the intramolecular charge transfer process [36]. In particular, for charge transfer purposes, the presence of unsaturated linkages (such as in ethylene and ethynylene) between the donor and acceptor units has been proven to be beneficial, as these linkages potentially reduce steric hindrance and enhance co-planarity along the molecular skeleton [37]. In this context, 3,4-ethylenedioxythiophene (EDOT) has a prominent position in the category of extended π-donors, because of its unique combination of strong electron donor properties and self-structuring effects related to the intramolecular non-covalent interactions between oxygen and sulfur [38]. These intramolecular interactions also exert a determining influence on the structure and electronic properties of donors incorporating EDOT units. However, so far, the polymeric counterpart of EDOT, i.e., poly (3, 4-ethylenedioxythiophene (PEDOT), has been extensively used for the purpose of solution processed organic electronics, while the application of EDOT is rare [39]. This motivated us to apply thiiophene and 3,4-ethylenedioxythiophene as donors in small molecule-based OTFTs.

**Scheme 1:**
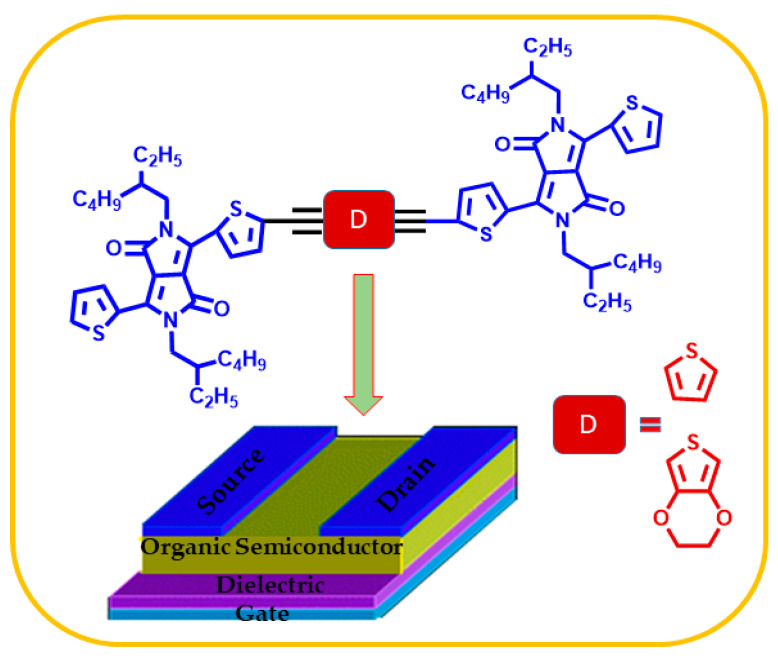
Schematic representation of A-π-D-π-A type conjugated small molecules, **TDPP-T** and **TDPP-EDOT**, for OTFTs.

Herein, we report two novel A-π-D-π-A type conjugated small molecules, namely **TDPP-T** and **TDPP-EDOT** (Scheme 1). In the two compounds, diketopyrrolopyrrole acts as an electron acceptor, while thiiophene and 3,4-ethylenedioxythiophene act as as electron donors. Incorporation of the rigid ethynylene bridge can increase conjugation, thus the absorption bands can be broadened to a longer wavelength region, further narrowing the band gap. The synthesized compound was investigated for its optical, electrochemical, thermal, and OTFT properties. The **TDPP-T** and **TDPP-EDOT** were confirmed by NMR spectra. The Figures S1–S4 in the supporting information represent the NMR spectra of the **TDPP-T** and **TDPP-EDOT.**

## 2. Materials and Methods

### 2.1. Materials

Sodium, FeCl_3_, 2-methyl-2-butanol, 2-ethyl hexyl bromide, Pd(PPh_3_)_4_, CuI, (i-Pr)_2_NH, ethynyltrimethylsilane, *N*-bromosuccinimide, 3,4-ethylenedioxythiophene, and 2,5-dibromothiophene were purchased from Sigma-Aldrich. DMF and 2-carbonitrile thiophene were purchased from Alfa Aesar Co. Tetrahydrofuran, dichloromethane, toluene, methanol, ethyl acetate, hexane, CHCl_3_, and potassium carbonate were purchased from Samchun Pure Chemicals. Tetrahydrofuran (THF), diethyl ether, toluene, and methylene chloride were used after distillation in the presence of sodium/benzophenone or calcium hydride under nitrogen gas.

### 2.2. Synthesis of Organic Compounds

#### 2.2.1. Synthesis of 3,6-Di(thiophen-2-yl)pyrrolo[3,4-c]pyrrole-1,4(2H,5H)-dione (**3**)

A mixture of sodium (7.39 g, 321.6 mmol), FeCl_3_ (0.31 g, 1.93 mmol), and dry 2-methyl-2-butanol (159 mL) was stirred at 120 °C for 3 h under an N_2_ atmosphere. Then, 2-carbonitrile thiophene (**2**) (14.99 mL, 160.8 mmol) was added and stirred for 30 min. Then, diethyl succinate (**1**) (8.4 mL, 9.40 mmol) in dry 2-methyl-2-butanol (66 mL) was added. After stirring the reaction mixture at 120 °C for 24 h, the contents in the flask were cooled to 50 °C, then acetic acid (55 mL) was added. After that, the mixture was refluxed for 1 h, cooled back to room temperature, and poured into methanol (50 mL). The precipitate was filtered and washed with water and methanol to afford (**3**) a dark red solid (10.5 g, 54%), which was used without further purification.

#### 2.2.2. Synthesis of 2,5-Bis(2-ethylhexyl)-3,6-di(thiophen-2-yl)-2,5-dihydropyrrolo[3,4-c]pyrrole-1,4-dione (**4**)

Compound **3** (21.75 g, 72.4 mmol) and potassium carbonate (40.01 g, 289.6 mmol) were refluxed in DMF (522 mL) at 120 °C for 1 h. Then, 2-ethyl hexyl bromide (48.936 g, 253.4 mmol) was added dropwise. The reaction mixture was stirred at 120 °C for 36 h and was then cooled to room temperature. CHCl_3_ (100 mL) and water (100 mL) were added and the layers were separated using a separating funnel. The organic layer was washed with brine, and the solvent was evaporated using a rotatory evaporator. The resulting solid was subjected to column chromatography (silica, eluent hexane/CH_2_Cl_2_, *v/v* 1/1) to afford (**4**) a red solid (1.5 g, 16%). ^1^H NMR (300 MHz, CDCl_3_, ppm): δ 8.91 (dd, *J* = 3.9, 1.2 Hz, 2H), 7.64 (dd, *J* = 5.1, 1.2 Hz, 2H), 7.28 (t, 2H), 4.04 (dd, *J* = 7.8, 3.6 Hz, 4H), 1.90–1.84 (m, 2H), 1.40–1.24 (m, 28); ^13^C NMR (75 MHz, CDCl_3_, ppm): δ 161.8, 140.4, 130.5, 129.8, 128.4, 107.9, 45.9, 39.1, 30.2, 28.4, 23.5, 23.1, 14.0, 10.5.

#### 2.2.3. Synthesis of 3-(5-Bromothiophen-2-yl)-2,5-bis(2-ethylhexyl)-6-(thiophen-2-yl)-2,5-dihydropyrrolo[3,4-c]pyrrole-1,4-dione (**5**)

To a compound **4** (2.5 g, 4.76 mmol) in chloroform (60 mL), NBS (0.68 g, 3.81 mmol) was added in small portions at room temperature and stirred the reaction mixture for overnight. Then water was added, and the reaction mixture was extracted three times with methylene chloride (50 mL). The collected organic layers were dried over MgSO_4_, filtered, and the solvent was evaporated under reduced pressure. The crude residue was purified by silica gel chromatography with methylene chloride/n-hexane, (1:1, *v/v*) as eluent to give **5** as a red solid. (0.8 g, 27%); ^1^H NMR (300 MHz, CDCl_3_, ppm): δ 8.92 (dd, *J* = 3.9, 1.2 Hz, 2H), 7.64 (dd, *J* = 4.8, 1.2 Hz, 2H), 7.29 (dd, *J* = 5.1, 3.9 Hz, 2H), 4.06–4.02 (m, 4H), 1.89–1.86 (m, 2H), 1.42–1.19 (m, 20H), 0.91–0.84 (m, 14H); ^13^C NMR (75 MHz, CDCl_3_, ppm): δ 161.6, 161.4, 140.9, 138.9, 135.6, 135.1, 131.4, 131.3, 130.9, 129.8, 128.5, 108.1, 107.8, 39.1, 39.1, 30.2, 28.3, 23.6, 23.5, 23.1, 14.0, 10.5.

#### 2.2.4. Synthesis of 2,5-Bis(2-ethylhexyl)-3-(thiophen-2-yl)-6-(5-((trimethylsilyl)ethynyl)thiophen-2-yl)-2,5-dihydropyrrolo[3,4-c]pyrrole-1,4-dione (**6**)

Compound **5** (0.5 g, 0.83 mmol), Pd(PPh_3_)_4_ (19.2 mg, 0.0166 mmol), and CuI (0.9 mg, 0.005 mmol) were added to a dried two-neck flask. Degassed anhydrous toluene (20 mL) and (i-Pr)_2_NH (4.16 mL) were added, followed by the addition of ethynyltrimethylsilane (163 mg, 1.66 mmol). Then, the reaction mixture was stirred under a nitrogen atmosphere at 90 °C overnight. After cooling to room temperature, water was added, and the mixture was extracted three times with ethyl acetate. The organic layer was dried over MgSO_4_ and was filtered. The solvent was evaporated under reduced pressure to afford compound **6** (557 mg) in a 90% yield. The crude product was used for the next step without further purification. ^1^H NMR (300 MHz, CDCl_3_, ppm): δ 8.84 (dd, *J* = 3.9, 0.9 Hz, 1H), 8.71 (d, *J* = 4.2 Hz, 1H), 7.55 (dd, *J* = 5.1, 0.9 Hz, 1H), 7.23 (d, *J* = 4.2, 1H), 7.18 (t, *J* = 4.2 Hz, 1H), 3.95–3.88 (m, 4H), 1.78–1.76 (m, 2H), 1.30–1.14 (m, 18H), 0.82–0.75 (m, 12H), 0.21–0.19 (m, 9H), ^13^C NMR (75 MHz, CDCl_3_,ppm): δ 161.9, 161.8, 141.0, 139.5, 135.9, 135.2, 133.8, 131.1, 130.8, 130.0, 128.9, 128.8, 128.3, 109.1, 108.3, 104.2, 97.0, 46.3, 46.2, 39.3, 30.5, 30.4, 28.6, 23.8, 23.3, 14.3, 10.7.

#### 2.2.5. Synthesis of 2,5-Bis(2-ethylhexyl)-3-(5-ethynylthiophen-2-yl)-6-(thiophen-2-yl)-2,5-dihydropyrrolo[3,4-c]pyrrole-1,4-dione (**7**)

K_2_CO_3_ (173 mg, 1.25 mmol) was added to a stirred solution of compound **6** (550 mg, 1 mmol) in CH_2_Cl_2_ (44 mL) at room temperature, and the reaction mixture was further stirred for 30 min. Then, 10 mL of water was added, and the mixture was extracted three times with ethyl acetate. The organic layer was dried over MgSO_4_ and was filtered. The solvent was evaporated under reduced pressure. The crude product was purified by column chromatography with CH_2_Cl_2_/n-hexane (*v/v*, 1:3) as an eluent to afford compound **7** (417 mg) in a 95% yield. ^1^H NMR (400 MHz, CDCl3, ppm): δ 8.93 (d, *J* = 4 Hz, 1H), 8.80 (d, *J* = 4 Hz, 1H), 7.65 (d, *J* = 4 Hz, 1H), 7.38 (d, *J* = 4 Hz, 1H), 7.29 (d, *J* = 4 Hz, 1H), 4.04–3.97 (m, 4H), 3.58 (s, 1H), 1.86 (br, 2H), 1.38–1.32 (m, 16H), 0.90–0.86 (m, 12H); ^13^C NMR (75 MHz, CDCl_3_,ppm): δ 161.7, 161.5, 141.1, 139.0, 135.8, 134.7, 134.0, 131.0, 130.9, 129.9, 129.7, 128.5, 126.6, 109.0, 108.0, 85.2, 46.0, 45.9, 39.2, 39.1, 31.9, 30.2, 29.7, 29.3, 28.3, 28.3, 27.2, 25.5, 23.5, 23.1,22.7, 14.1, 14.0, 10.5.

#### 2.2.6. Synthesis of 5,7-Dibromo-2,3-dihydrothieno[3,4-b][1,4]dioxine (**9**)

**Scheme 2:**
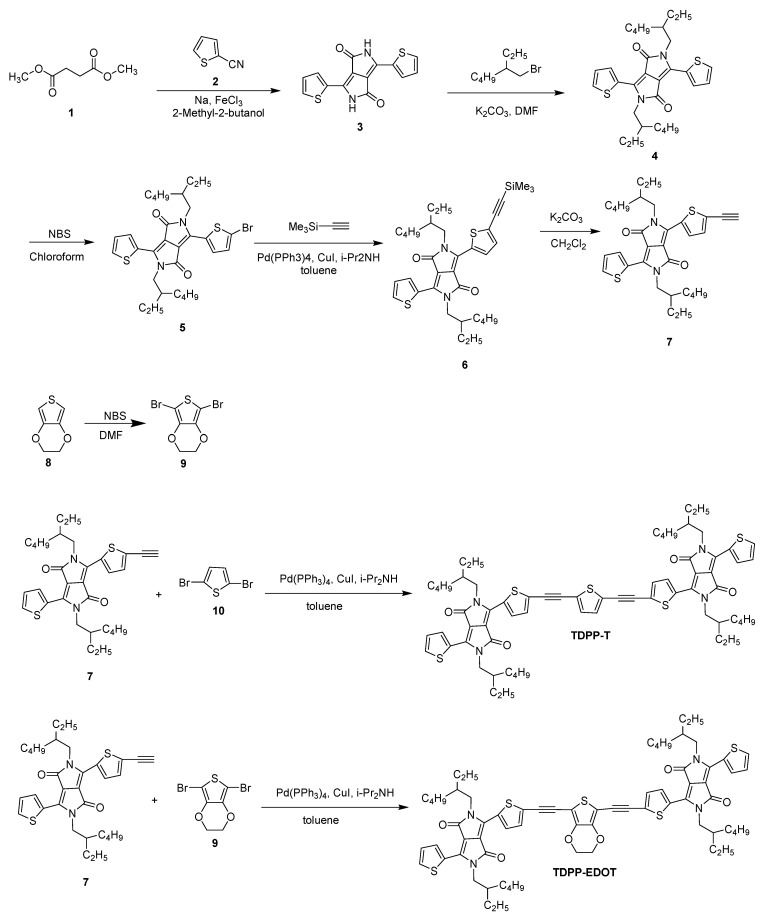
Synthesis of **TDPP-T** and **TDPP-EDOT**.

3,4-Ethylenedioxythiophene (**8**) (1.5 g, 10.55 mmol) was dissolved in 32 mL of DMF. *N*-Bromosuccinimide (3.94 g, 22.155 mmol) was dissolved in 32 mL DMF and was added to the reaction mixture dropwise at 0 °C. The solution was stirred at room temperature for 4 h under the exclusion of light. After the completion of the reaction, water was added and filtered. The precipitate was washed with water and the resulting precipitate was dried under a xzvacuum to afford compound **9** (2.1 g, 66%) (Scheme 2). ^1^H NMR (300 MHz, CDCl_3_, ppm): δ 4.2 (s, 2H); ^13^C NMR (75 MHz, CDCl_3_, ppm): δ 139.7, 85.6, 65.0.

#### 2.2.7. Synthesis of 6,6′-((Thiophene-2,5-diylbis(ethyne-2,1-diyl))bis(thiophene-5,2-diyl))bis(2,5-bis(2-ethylhexyl)-3-(thiophen-2-yl)-2,5-dihydropyrrolo[3,4-c]pyrrole-1,4-dione) (**TDPP-T**)

Compound **7** (0.8 g, 1.45 mmol), Pd(PPh_3_)_4_ (34 mg, 0.029 mmol), and CuI (1.7 mg, 0.0087 mmol) were loaded into a dried two-neck flask. Degassed anhydrous toluene (29 mL) and (i-Pr)_2_NH (7 mL) were added, followed by the addition of 2,5-dibromothiophene (**10**) (140 mg, 0.58 mmol). Then, the reaction mixture was heated to 90 °C and stirred overnight. After cooling to room temperature, water was added and the mixture was extracted three times with ethyl acetate. The organic phase was dried over MgSO_4_ and filtered. Then, the filtrate was concentrated under reduced pressure. The crude product was purified via column chromatography with CH_2_Cl_2_/n-hexane (*v/v*, 2:1) as an eluent to afford **TDPP-T** (397 mg) in a 58% yield (Scheme 2). ^1^H NMR (300 MHz, CDCl_3_, ppm): δ 8.96 (dd, *J* = 3.9, 1.2 Hz, 2H), 8.88 (d, *J* = 4.2 Hz, 2H), 7.67 (dd, *J* = 5.1, 1.2 Hz, 2H), 7.42 (d, *J* = 4.2 Hz, 2H), 7.30 (dd, *J* = 5.1, 3.9 Hz, 2H), 7.26 (s, 2H), 4.07–4.02 (m, 8H), 1.91–1.87 (m, 4H), 1.43–1.26 (m, 32H), 0.95–0.88 (m, 24H); ^13^C NMR (75 MHz, CDCl_3_, ppm): δ 161.6, 161.5, 140.9, 138.9, 135.8, 135.2, 133.4, 132.8, 131.4, 131.0, 129.8, 128.5, 127.1, 124.7, 109.0, 108.0, 90.1, 87.9, 46.1, 46.0, 39.2, 39.1, 30.2, 30.1, 28.4, 23.5, 23.1, 14.1, 14.0, 10.5.

#### 2.2.8. Synthesis of 6,6′-(((2,3-Dihydrothieno[3,4-b][1,4]dioxine-5,7-diyl)bis(ethyne-2,1-diyl))bis(thiophene-5,2-diyl))bis(2,5-bis(2-ethylhexyl)-3-(thiophen-2-yl)-2,5-dihydropyrrolo[3,4-c]pyrrole-1,4-dione) (**TDPP-EDOT**)

Compound **7** (0.65 g, 1.175 mmol), Pd(PPh_3_)_4_ (27 mg, 0.029 mmol), and CuI (1.3 mg, 0.007 mmol) were loaded into a dried two-neck flask. Degassed anhydrous toluene (23 mL) and (i-Pr)_2_NH (6 mL) were added, followed by the addition of compound **9** (0.14 g, 0.47 mmol). Then, the reaction mixture was heated to 90 °C and stirred overnight. After cooling to room temperature, water was added and the mixture was extracted three times with ethyl acetate. The organic phase was dried over MgSO_4_ and was filtered. Then, the filtrate was concentrated under reduced pressure. The crude product was purified via column chromatography with CH_2_Cl_2_/n-hexane (*v/v*, 2:1) as an eluent to afford **TDPP-EDOT** (0.35 g) in a 60% yield. ^1^H NMR (300 MHz, CDCl_3_, ppm): δ 8.95 (dd, *J* = 3.9, 1.2 Hz, 2H), 8.89 (d, *J* = 4.2 Hz, 2H), 7.66 (dd, *J* = 5.1, 1.2 Hz, 2H), 7.41 (d, 4.2Hz, 2H), 7.23 (dd, *J* = 5.1, 3.9 Hz, 2H), 4.38 (s, 4H), 4.07–4.01 (m, 8H), 1.91–1.87 (m, 4H), 1.42–1.23 (m, 32H), 0.94–0.85 (m, 24H); ^13^C NMR (75 MHz, CDCl_3_, ppm): δ 161.7, 161.6, 143.9, 140.8, 139.1, 135.7, 135.3, 133.3, 131.2, 130.9, 129.8, 128.5, 127.4, 108.9, 108.1, 100.3, 90.4, 87.8, 64.9, 46.1, 46.0, 39.1, 39.1, 30.2, 30.1, 28.4, 23.5, 23.1, 14.0, 10.5.

### 2.3. Measurements

The ^1^H and ^13^C NMR spectra were measured using an NMR spectrometer (Bruker DRX-300, Berlin, Germany). The values of the chemical shift were stated in δ units (ppm). Mass spectral investigation was conducted on a JMS-700, JEOL. The absorption studies were attained by using a UV–VIS spectrophotometer (Shimadzu, UV-1065PC, Tokyo, Japan). Cyclic voltammogram examinations were conducted using an epsilon E_3_ at room temperature (RT) in a 0.1M tetrabutylammonium perchlorite solution in chloroform under an N_2_ atmosphere at a 50 mV/s scan rate. The Ag/AgCl and Pt wire served as the reference and counter electrodes, respectively. The thermal properties were examined using a thermogravimetric analysis (TGA) and differential scanning calorimeter (DSC) (Mettler Toledo AG, Analytical, Schwerzenbach, Switzerland). A DSC analysis was conducted under an N_2_ atmosphere, at temperature ranges from 0–400 °C at a heating rate of 10 °C/min. The thermogravimetric analysis (TGA) was measured under an N_2_ atmosphere, at a heating rate from room temperature to 800 °C, at a of 10 °C/min using a thermogravimetric analyzer. The OFET top-contact and bottom-gate were made-up on a common gate of highly n-doped silicon with a thick, thermally grown 300 nm SiO_2_ dielectric layer. Further modification of the dielectric SiO_2_ layer was carried out using a self-assembled octyltrichlorosilane (OTS) monolayer. The solution comprising the semiconductor was spin-coated at 2000 rpm from 0.5–1 wt.% in chloroform to attain the thin film. Gold source and drain electrodes were evaporated on top of the semiconductor layer. Entire the investigation procedure was carried out by a home-built lab-view program combined with Keithley electronic units in a nitrogen atmosphere.

## 3. Results and Discussions

### 3.1. Optical and Electrochemical Properties of the ***TDPP-T*** and ***TDPP-EDOT***

**Figure 1:**
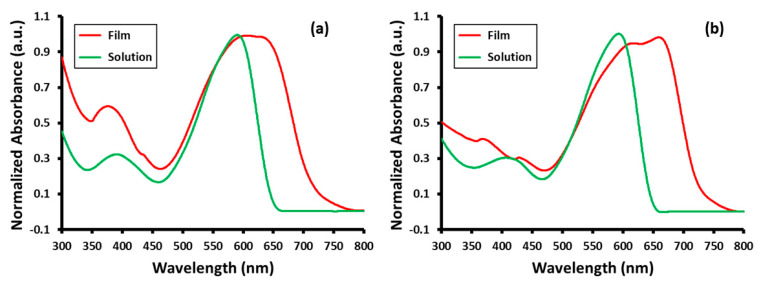
UV–VIS absorption spectra of (**a**) **TDPP-T** and (**b**) **TDPP-EDOT** in chloroform solution and thin films.

The optical properties of **TDPP-T** and **TDPP-EDOT** were investigated using UV–VIS absorption spectroscopy. The UV–VIS absorption spectra of **TDPP-T** and **TDPP-EDOT** in a chloroform solution and in thin films are shown in Figure 1. The resulting data are summarized in Table 1. The UV–VIS absorption spectra of the two compounds exhibited two absorption bands in both the solution and film states. The higher wavelength band corresponds to intramolecular charge transfer (ICT) between donor and acceptor groups, which is typical in donor–acceptor-based compounds. In solution, **TDPP-T** showed absorption peaks at 391 and 591 nm, whereas **TDPP-EDOT** showed at 410 and 594 nm. In the film state, the absorption peaks for **TDPP-T** and **TDPP-EDOT** were observed at 376, 607, and 630 nm, and 370, 415, and 663 nm, respectively. In both compounds the ICT band in the film state were broad and red-shifted when compared with the solution state because of the intermolecular interactions in the film state. The optical band gap energy calculated for **TDPP-T** and **TDPP-EDOT** from the thin film state was 1.73 eV and 1.70 eV, respectively. In TDPP-EDOT, 3,4-ethylenedioxythiophene had a strong electron-releasing group compared with the thiophene in TDPP-T [40]. Because of the enhanced electron-releasing effect, it increased the HOMO level and resulted in a reduction of the band gap. The donor–acceptor character contributed to a narrowing of the band gap energy between the HOMO and LOMO levels.

**Figure 2:**
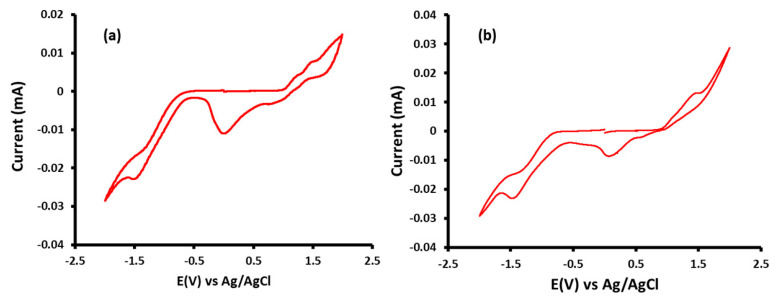
Cyclic voltammogram curves of (**a**) **TDPP-T** and (**b**) **TDPP-EDOT**.

To investigate the electrochemical properties of **TDPP-T** and **TDPP-EDOT**, cyclic voltammetry (CV) measurements were employed, and the results are summarized in Table 1. The cyclic voltammetry (CV) of **TDPP-T** and **TDPP-EDOT** are shown in Figure 2. Both **TDPP-T** and **TDPP-EDOT** showed oxidation and reduction waves. The HOMO energy levels of **TDPP-T** and **TDPP-EDOT** were calculated from the onset oxidation potentials to be −5.44 eV and −5.31 eV, respectively. In **TDPP-T**, the donor thiophene group was less electron releasing compared with 3,4-ethylenedioxythiophene in **TDPP-EDOT**, and, as a result, **TDPP-T** had a deeper HOMO level. The LUMO energy levels were evaluated to be −3.71 eV and −3.61 eV, respectively, by using the HOMO values and optical band gaps (LUMO = E_g_^opt^ + HOMO).

### 3.2. Thermal Properties of the ***TDPP-T*** and ***TDPP-EDOT***

**Figure 3:**
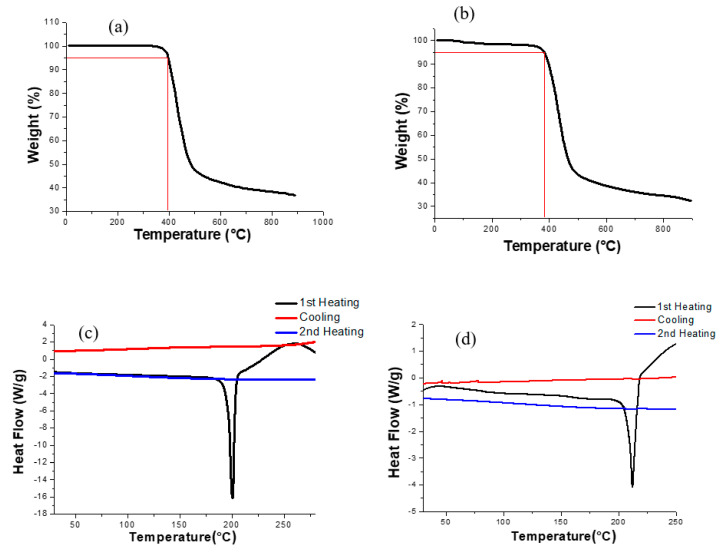
TGA curves of (**a**) **TDPP-T** and (**b**) **TDPP-EDOT**, DSC curves of (**c**) **TDPP-T** and (**d**) **TDPP-EDOT**.

The thermal properties of **TDPP-T** and **TDPP-EDOT** were investigated using thermo gravimetric analysis (TGA) and differential scanning calorimetry (DSC). The TGA and DSC curves are shown in Figure 3. The thermal properties are summarized in Table 1. The thermal properties of the materials used as the organic thin film transistor have a major influence on the stability and lifetime of the resulting device. The decomposition temperatures (5% weight loss, T_d_) for compounds **TDPP-T** and **TDPP-EDOT** were observed at 395 and 383 °C, respectively, indicating that the compounds were thermally stable and could be used for OTFTs. The thermal decomposition temperature at 5% wt. loss for TDPP-T was further compared with TDPP-EDOT. Hence, TDPP-T was more thermally stable than the TDPP-EDOT. In the DSC analysis, the melting temperatures (T_m_) of **TDPP-T** and **TDPP-EDOT** were observed at 201 and 211 °C, respectively.

### 3.3. OTFT Characterization of the ***TDPP-T*** and ***TDPP-EDOT***

**Figure 4:**
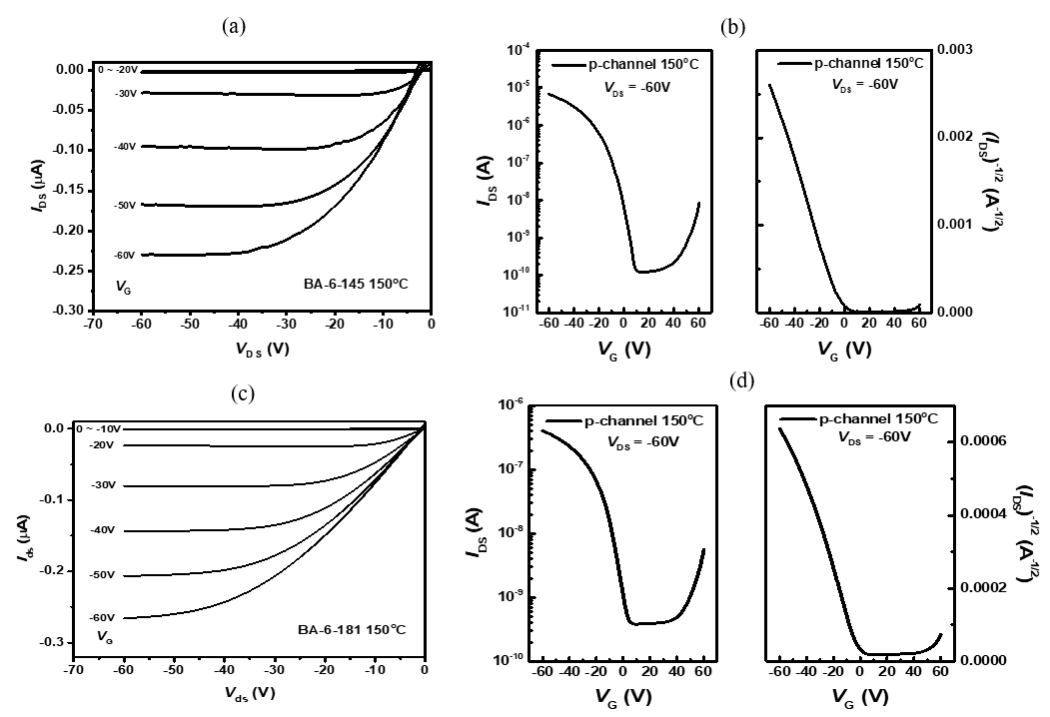
OTFT field-effect transistor characteristics. Output curves of (**a**) **TDPP-T** and (**c**) **TDPP-EDOT**. Transfer curves of (**b**) **TDPP-T** and (**d**) **TDPP-EDOT**.

OTFT devices were fabricated using bottom gate and top contact (BGTC) configuration. The OTFT films were prepared using the SiO_2_ insulator while octyltrichlorosilane (OTS) was used for the surface treatment. Active layers were deposited by spin-coating a solution of semiconductors (5 mg mL^−1^ in CHCl_3_) at 1500 rpm. The thickness of the thin film was 30 nm. The source and drain electrodes were deposited by thermal evaporation of Au through a shadow mask. The Au thickness was 100 nm. The hole mobility (μ) was determined using the following equation in the saturation regime: Ids = (WCi/2L) × μ × (Vgs − VT)^2^, where Ci is the capacitance per unit area of the SiO_2_ dielectric (Ci = 15 nF cm^−2^), Ids is the drain–source current, Vgs is the gate voltage, and VT is the threshold voltage. The OTFT devices were tested using a Keithley 4200 semiconductor characterization system under a nitrogen atmosphere. The voltage–current graphs of the OTFT devices are shown in Figure 4. The OTFT characteristic properties are summarized in Table 2. Both **TDPP-T** and **TDPP-EDOT** showed a p-type mobility. In the as cast film, **TDPP-T** and **TDPP-EDOT** showed a hole mobility of 5.44 × 10^−6^ cm^2^ V^−1^ s^−1^ and 4.13 × 10^−6^ cm^2^ V^−1^ s^−1^, respectively. After thermal annealing, the mobilities increased because the crystallinity was enhanced. The optimal annealing temperatures for **TDPP-T** and **TDPP-EDOT** were 150 °C. At this temperature, the devices showed a hole mobility of 3.11 × 10^−4^ cm^2^ V^−1^ s^−1^ and 2.63 × 10^−4^ cm^2^ V^−1^ s^−1^, respectively. The large threshold voltage of 42.6 V for the as-cast **TDPP-EDOT** devices is due to the poor interface conditions. Thermal annealing causes the modification the Au/SiO_2_ interface, which further leads to a reduction of the threshold voltage [41].

**Figure 5:**
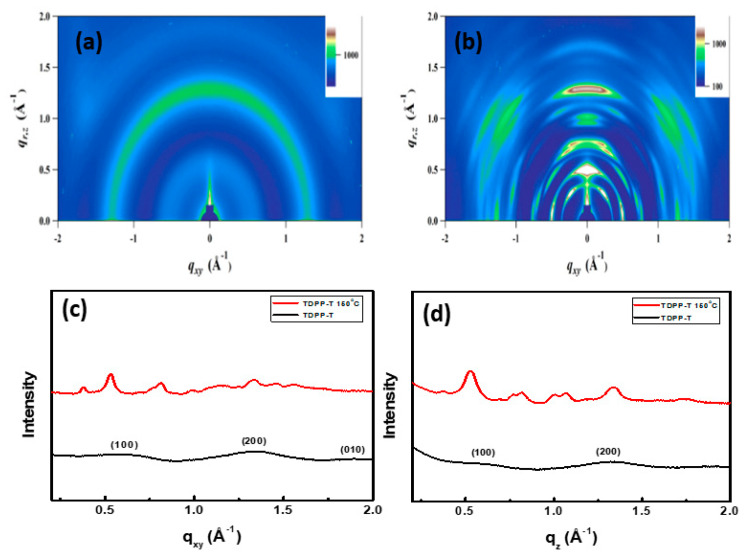
Two-dimensional grazing incidence X-ray diffraction images of **TDPP-T** (**a**) without annealing and (**b**) with being annealed at 150 °C, corresponding line-cuts along the (**c**) q_xy_ and (**d**) q_z_ plane.

**Figure 6:**
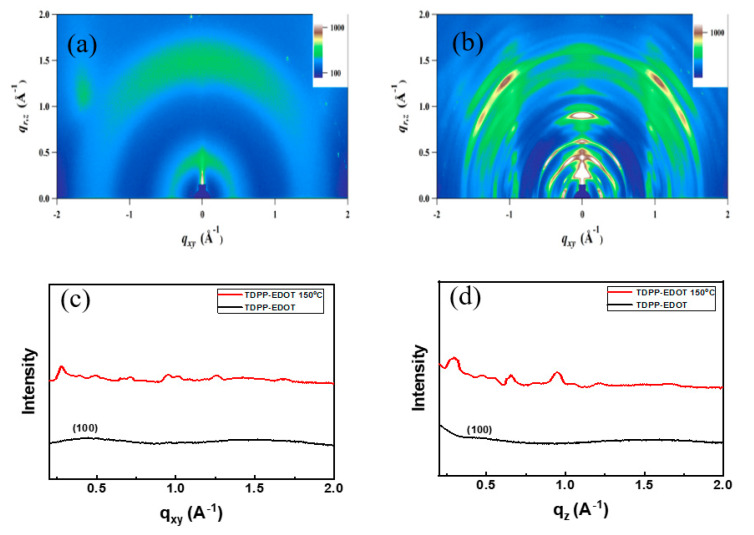
Two-dimensional grazing incidence X-ray diffraction images of **TDPP-EDOT** (**a**) without annealing and (**b**) with being annealed at 150 °C, corresponding line-cuts along the (**c**) q_xy_ and (**d**) q_z_ plane.

The crystallinity and molecular ordering of thin films were investigated using X-ray diffraction (2D-GIXRD) patterns. The 2D-GIXRD patterns of **TDPP-T** and **TDPP-EDOT** are shown in Figure 5 and Figure 6. The 2D-GIXRD patterns of **TDPP-T** and **TDPP-EDOT** were analyzed for the films as cast at 150 °C temperatures. In both compounds, **TDPP-T** and **TDPP-EDOT**, compared with the as cast film, thermal annealing at 150 °C increased the crystallinity, thus leading to an increase in mobility. The low mobilities were due to the poor crystallinity or low morphological orientation of the organic molecules. The mobility can be improved by using additives [42,43,44,45]. The organic molecules blended with additives cause phase segregation and increased crystallinity. Thus, the blended films will give allow for improved OTFT performances.

## 4. Conclusions

The donor–acceptor-based small molecule, **TDPP-T** and **TDPP-EDOT** were designed and synthesized. The band gap energy between the HOMO and LUMO was decreased due to the donor–acceptor interactions in the molecule. The alkyl chains promoted the good solubility of the compound in common organic solvents. **TDPP-T**- and **TDPP-EDOT**-based OTFT devices after thermal annealing at 150 °C have shown hole mobility of 3.11 × 10^−4^ cm^2^ V^−1^ s^−1^ and 2.63 × 10^−4^ cm^2^ V^−1^ s^−1^, respectively.

## Figures and Tables

**Table 1:** Optical, electrochemical, and thermal properties of **TDPP-T** and **TDPP-EDOT**.

Compound	λ_abs_/nm		E_g_^a^ (eV)	HOMO (eV) ^b^	LUMO (eV) ^c^	T_d_ (°C) ^d^	T_m_ (°C) ^e^
	Solution	Film					
**TDPP-T**	391, 591	376, 607,630	1.73	−5.44	−3.71	395	201
**TDPP-EDOT**	410, 594	370, 415,663	1.70	−5.31	−3.61	383	211

^a^ Calculated from the absorption edge wavelength. ^b^ Calculated from the onset of the oxidation potential. ^c^ Calculated from the HOMO level-band gap. ^d^ Decomposition temperature at 5% weight loss. ^e^ Tm, endothermic melting point.

**Table 2:** OTFT device performance of **TDPP-T** and **TDPP-EDOT**.

	*µ*_AVG_ (cm^2^/Vs)		*V*_TH_ (V)		On/Off Ratio	
	TDPP-T	TDPP-EDOT	TDPP-T	TDPP-EDOT	TDPP-T	TDPP-EDOT
As cast	(5.44 ± 0.11) × 10^−6^	(4.13 ± 0.16) × 10^−6^	−5.0	42.6	13.2	3.5
Ann 100 °C	(3.61 ± 0.08) × 10^−5^	(1.26 ± 0.06) × 10^−5^	−1.5	−2.5	23.1	73.4
Ann 150 °C	(3.11 ± 0.08) × 10^−4^	(2.63 ± 0.12) × 10^−4^	−11.5	1.4	326	1060

## Data Availability

Data are contained within the article or supplementary file.

## Supplementary Materials

The following are available online at https://www.mdpi.com/article/10.3390/mi12070817/s1. Figure S1. ^1^H NMR (300 MHz, CDCl_3_) spectrum of compound **TDPP-T**. Figure S2. ^13^C NMR (75 MHz, CDCl_3_) spectrum of compound **TDPP-T**. Figure S3. ^1^H NMR (300 MHz, CDCl_3_) spectrum of compound **TDPP-EDOT**. Figure S4. ^13^C NMR (75 MHz, CDCl_3_) spectrum of compound **TDPP-EDOT**.
