# Supplementary material for: A-π-D-π-A-Based Small Molecules for OTFTs Containing Diketopyrrolopyrrole as Acceptor Units
Source: Micromachines (Basel). 2021 Jul 13;12(7):817. doi: 10.3390/mi12070817 (PMC8304449; doi:10.3390/mi12070817)
Supplement: Supplementary file 1 [file micromachines-12-00817-s001.zip › micromachines-1246668-supplementary.pdf]

micromachines

Supplementary File

A-π-D-π-A-Based Small Molecules for OTFTs Containing Diketopyrrolopyrrole as Acceptor Units

Baji Shaik [1], Mujeeb Khan [2], Mohammed Rafi Shaik [2,*], Mohammed A.F. Sharaf [3], Doumbia Sekou [4] and Sang-Gyeong Lee [1,*]

[1] Department of Chemistry, Research Institute of Natural Science (RINS), Graduate School for Molecular Materials and Nanochemistry, Gyeongsang National University, Jinju 660-701, South Korea; shaikbaji2@gmail.com
[2] Department of Chemistry, College of Science, King Saud University, P.O. Box 2455, Riyadh 11451, Saudi Arabia; kmujeeb@ksu.edu.sa
[3] Department of Industrial Engineering, College of Engineering, King Saud University, P.O. Box 800, Riyadh 11421, Saudi Arabia; mfsharaf@ksu.edu.sa
[4] Department of Agricultural Extension and Rural Society, College of Food and Agriculture Sciences, King Saud University, P.O. Box 2460, Riyadh 11451, Saudi Arabia; 442106474@student.ksu.edu.sa
* Correspondence: mrshaik@ksu.edu.sa (M.R.S.); leesang@gnu.ac.kr (S.-G.L.); Tel.: +966-11-4670439 (M.R.S.)

Citation: Shaik, B.; Khan, M.; Shaik, M.R.; Sharaf, M.A.F.; Sekou, D.; Lee, S.-G. A-π-D-π-A Based Small Molecules for OTFTs Containing Diketopyrrolopyrrole as Acceptor Units. *Micromachines* **2021**, *12*, 817. https://doi.org/10.3390/mi12070817

Academic Editors: Hocheon Yoo and Joohoon Kang

Received: 19 May 2021
Accepted: 11 July 2021
Published: 13 July 2021

Publisher's Note: MDPI stays neutral with regard to jurisdictional claims in published maps and institutional affiliations.

Copyright: © 2021 by the authors. Licensee MDPI, Basel, Switzerland. This article is an open access article distributed under the terms and conditions of the Creative Commons Attribution (CC BY) license (http://creativecommons.org/licenses/by/4.0/).

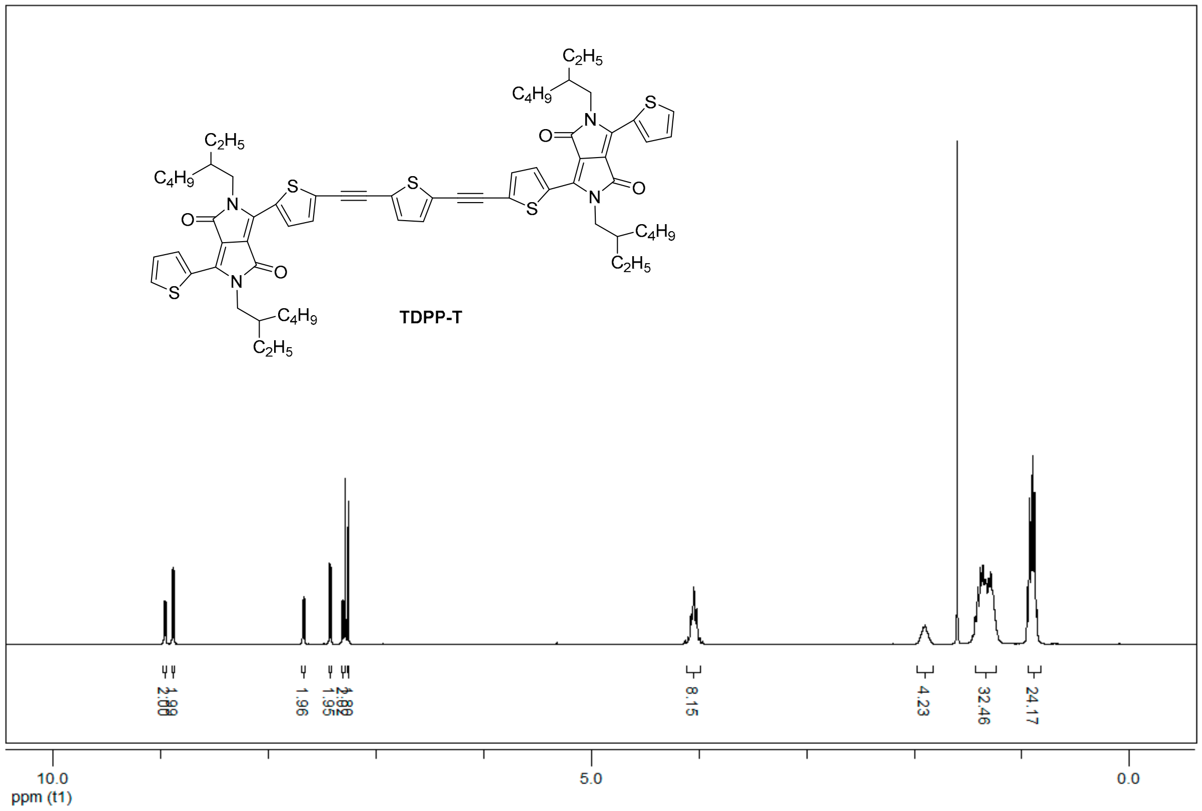

Figure S1. ^{1}H NMR (300 MHz, $CDCl_3$) spectrum of compound **TDPP-T**.

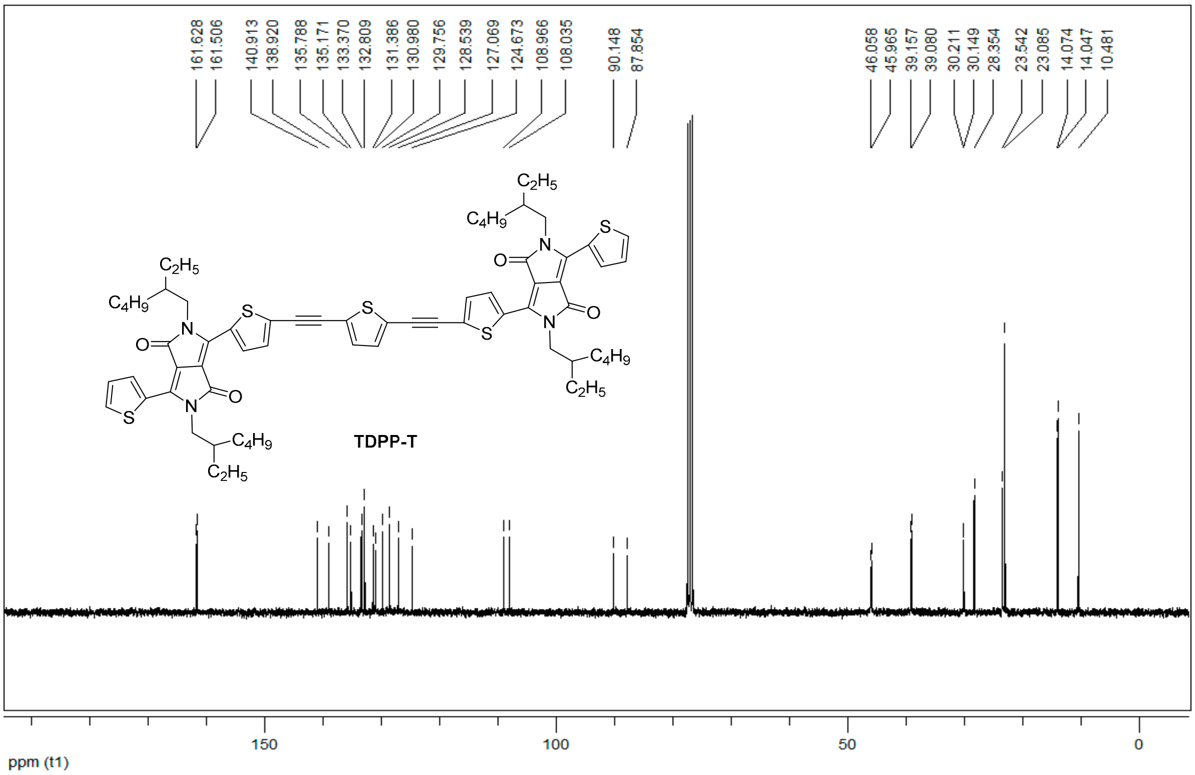

Figure S2. ^{13}C NMR (75 MHz, $CDCl_3$) spectrum of compound **TDPP-T**.

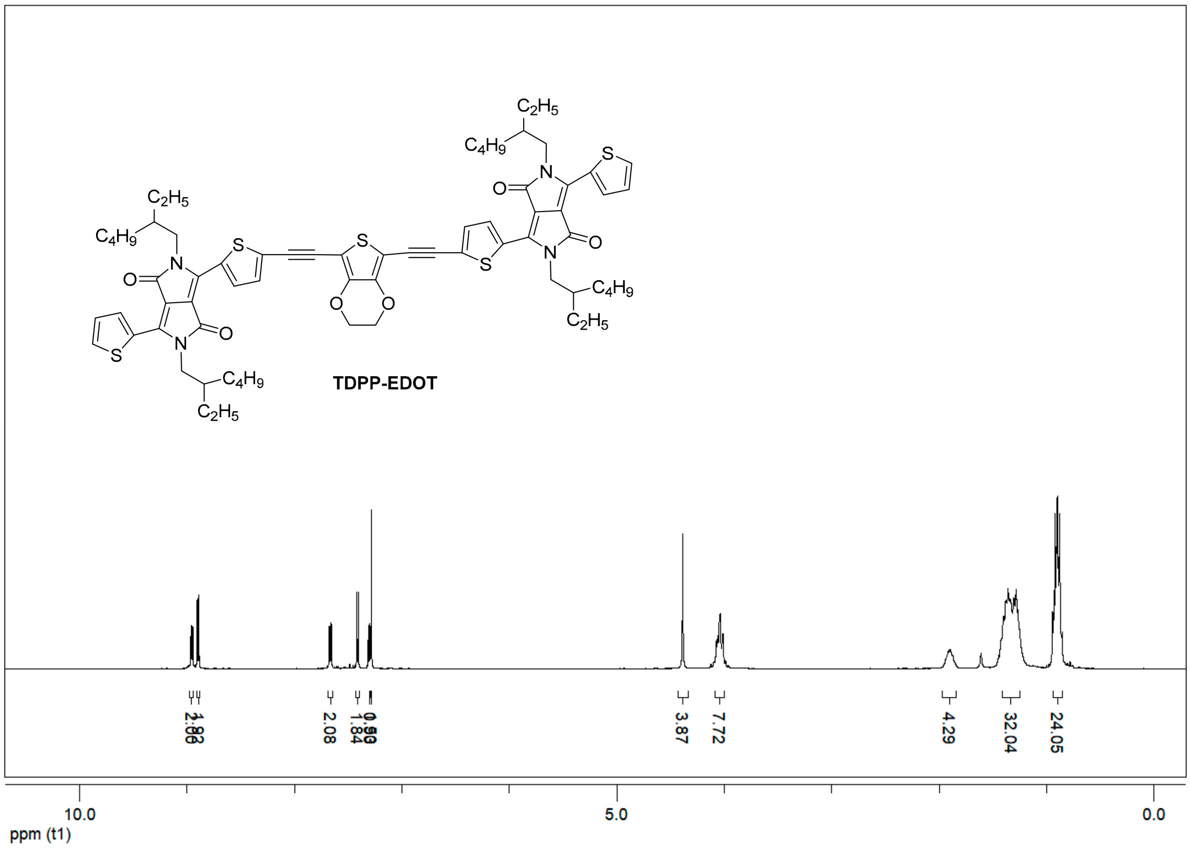

Figure S3. ^{1}H NMR (300 MHz, $CDCl_3$) spectrum of compound **TDPP-EDOT**.

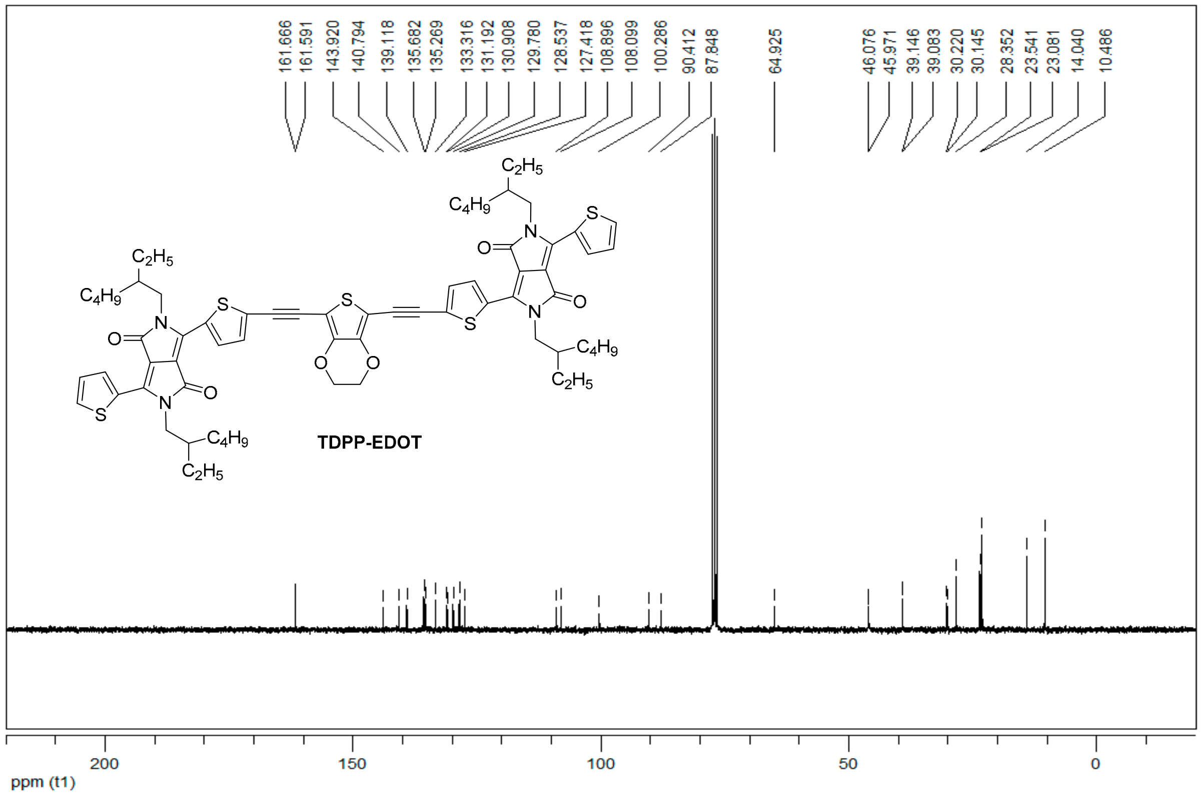

Figure S4. ^{13}C NMR (75 MHz, $CDCl_3$) spectrum of compound **TDPP-EDOT**.
